# Automated analysis of calcium spiking profiles with CaSA software: two case studies from root-microbe symbioses

**DOI:** 10.1186/1471-2229-13-224

**Published:** 2013-12-26

**Authors:** Giulia Russo, Salvatore Spinella, Eva Sciacca, Paola Bonfante, Andrea Genre

**Affiliations:** 1 Dipartimento di Scienze della Vita e Biologia dei Sistemi, Università di Torino, Viale P.A. Mattioli 25, 10125 Torino, Italy; 2 Dipartimento di Informatica, Università di Torino, C.So Svizzera, 185, 10149 Torino, Italy

**Keywords:** Arbuscular mycorrhiza, Calcium signaling, Medicago truncatula, Nitrogen fixation, Phosphate, Plant-microbe interactions, Symbiosis, Automated data analysis

## Abstract

**Background:**

Repeated oscillations in intracellular calcium (Ca^2+^) concentration, known as Ca^2+^ spiking signals, have been described in plants for a limited number of cellular responses to biotic or abiotic stimuli and most notably the common symbiotic signaling pathway (CSSP) which mediates the recognition by their plant hosts of two endosymbiotic microbes, arbuscular mycorrhizal (AM) fungi and nitrogen fixing rhizobia. The detailed analysis of the complexity and variability of the Ca^2+^ spiking patterns which have been revealed in recent studies requires both extensive datasets and sophisticated statistical tools.

**Results:**

As a contribution, we have developed automated Ca^2+^ spiking analysis (CaSA) software that performs i) automated peak detection, ii) statistical analyses based on the detected peaks, iii) autocorrelation analysis of peak-to-peak intervals to highlight major traits in the spiking pattern.

We have evaluated CaSA in two experimental studies. In the first, CaSA highlighted unpredicted differences in the spiking patterns induced in *Medicago truncatula* root epidermal cells by exudates of the AM fungus *Gigaspora margarita* as a function of the phosphate concentration in the growth medium of both host and fungus. In the second study we compared the spiking patterns triggered by either AM fungal or rhizobial symbiotic signals. CaSA revealed the existence of different patterns in signal periodicity, which are thought to contribute to the so-called Ca^2+^ signature.

**Conclusions:**

We therefore propose CaSA as a useful tool for characterizing oscillatory biological phenomena such as Ca^2+^ spiking.

## Background

As a ubiquitous second messenger, calcium (Ca^2+^) mediates multiple signal transduction pathways in diverse types of plant responses to biotic and abiotic stimuli [1,2]. Transient variations in calcium concentration occurring in the cytosol, nucleus and/or other compartments of the plant cell are believed to transduce extracellular signals into appropriate cellular responses [3]. These range from changes in turgor pressure as in stomatal guard cells [4,5], to the control of apical growth in pollen tubes [6] or the regulation of gene expression [7]. One of the most intriguing and well-studied types of Ca^2+^ signaling is the generation of repeated peaks in Ca^2+^ concentration that can persist for relatively long periods of time (minutes to hours) and is commonly referred to as “Ca^2+^ spiking” [1,4,8].

Ca^2+^ spiking is characterized by distinctive features such as peak shape, amplitude, frequency and regularity, certain of which may confer a degree of specificity in the respective stimulus-response coupling. Amongst the best studied examples, the regulation of stomatal closure was demonstrated to be based on peak frequency [5], and in the case of host-endosymbiont signaling differences in Ca^2+^ spiking regularity may be important in the recognition of the bacterial or fungal partner [9,10].

Unfortunately, the detailed characterization of such traits, which contribute to the so-called ‘calcium signature’ of the respective signal transduction pathway [11], is complicated by the intrinsic cell-to-cell variability of the oscillatory response [9,10,12,13]. In addition, background noise often masks ‘true’ peaks with clusters of smaller random oscillations. For these reasons, Ca^2+^ spiking analyses always require large data-sets before general trends can be clearly established. As a result, manual large scale analyses and the application of statistical tools is often very time consuming and can lead to the introduction of human operator biases. Fully standardizing the analysis is a therefore a prerequisite for correctly analyzing Ca^2+^ spiking signatures.

Indeed, many analytical methods have been developed in other areas, such as neuroscience, to analyze and classify the repeated oscillations in a biological parameter such as membrane potential (e.g. [12-15]). Nevertheless, such analytical processes largely relate peak identification to their regular frequency. Since an intrinsic trait in symbiotic Ca^2+^ spiking signals in plants is a variable extent of irregularity [9,12], such analyses based on peak prediction could not be successfully applied to our experimental data. We therefore chose to try a different approach and analytically characterize the phenomenon by identifying each spike based on its shape and how much this differs from background noise oscillations.

With this aim, we have prototyped original software that performs an automated Ca^2+^ spiking analysis (CaSA) based on our recent studies where a prototypal computational model was formalized to simulate Ca^2+^ spiking dynamics [16]. The CaSA software that we present here can quickly and reliably analyze large data sets, independently of the instrument or method used for recording the biological signal, and perform automatic peak detection and several statistical analyses. A pipeline chart schematizing data preparation and CaSA usage can be found in Additional file 1. The CaSA software is coded in GNU OCTAVE [17], a high-level interpreted language, and can be run on GNU/Linux systems.

To evaluate the software reliability we have applied CaSA to nuclear Ca^2+^ spiking profiles obtained with root epidermal cells of the legume *Medicago truncatula*. We have chosen two case studies, both based on the induction of nuclear Ca^2+^ spiking by signals released from two symbiotic root-interacting microbes: the arbuscular mycorrhizal (AM) fungus *Gigaspora margarita* and the symbiotic nitrogen-fixing (SNF) bacterium *Sinorhizobium meliloti*. Legumes can establish mutualistic interactions with both types of root symbionts. The AM symbiosis develops inside the root tissues, where living cortical cells are colonized by specialized hyphal structures called arbuscules. In contrast, SNF rhizobia colonize specialized root-derived organs, called root nodules, where they convert atmospheric nitrogen into ammonia, that can be later assimilated into organic compounds that can be assimilated by the host [18].

For both AM and SNF, initial stages of the symbiotic association require reciprocal recognition between the host plant and the respective microbe. Rhizobia are identified by the host plant through the perception of secreted lipochito-oligosaccharides (LCOs) known as “Nod factors” [19]. Recent evidence suggests that the molecules mediating the recognition of AM fungi may also be based on chito-oligosaccharides, either LCOs (so-called “Myc LCOs”) [20] or undecorated, short-chain chito-oligosaccharides such as chito-tetraose (CO4) [13]. Downstream of their perception, these chitinaceous signals are transduced by a common symbiotic signalling pathway (CSSP) involving a common subset of plant proteins. One of these proteins, localized in the nucleoplasm, is a calcium- and calmodulin-dependent kinase (MtDMI3 in *M. truncatula*) [21]. This key component of the CSSP is believed to interpret the respective nuclear Ca^2+^ spiking response which is activated following symbiotic signal perception [10,22,23].

AM fungi are beneficial to the plant since thay are able to extract inorganic phosphate (Pi) and other mineral nutrients from the soil with a higher efficiency compared to the non-symbiotic root system [24]. Consistent with this, high Pi availability in the soil is well known to limit the overall level of root colonization by AM fungi [25-27], although the underlying molecular mechanisms are only starting to emerge [28]. Furthermore, Pi availability has been shown to reduce the plant biosynthesis and secretion of strigolactones, key regulators of presymbiotic fungal development (for a review, [29]).

In our first case study we have investigated whether the Pi concentration in the growth medium also impacts the earliest plant responses to AM fungal signals by monitoring nuclear Ca^2+^ oscillations in the host epidermis. Our results using CaSA analysis have allowed us to detect a significant inhibition of presymbiotic signaling in response to high Pi levels.

How nuclear Ca^2+^ spiking specifically encodes AM- or SNF-related signals within the CSSP, and how these are then decoded by DMI3 to activate the appropriate downstream responses remains unclear. One possibility is that the pattern of the spiking profiles contains the encoded information in the form of a specific Ca^2+^ signature. For this reason the spiking profiles induced in legumes by both AM fungi and rhizobia have been the subject of several studies [9,12]. In our second case study we have applied the CaSA software to analyze the spiking profiles induced by either Nod factor or the putative AM CO4 signals in the epidermis of *M. truncatula* roots. Our results underline highly significant differences in the regularity of peak-to-peak intervals, suggesting that this trait could be part of the signature used by the plant cell to discriminate between the two signals.

## Results

### The CaSA software

CaSA software comprises a workflow of programming modules to analyze time-series data. It was prototyped in the GNU Octave language on the GNU/Linux platform. To run the GNU Octave software, the Gnuplot graphic utility and Zenity/GTK + tools are required.

**Figure 1:**
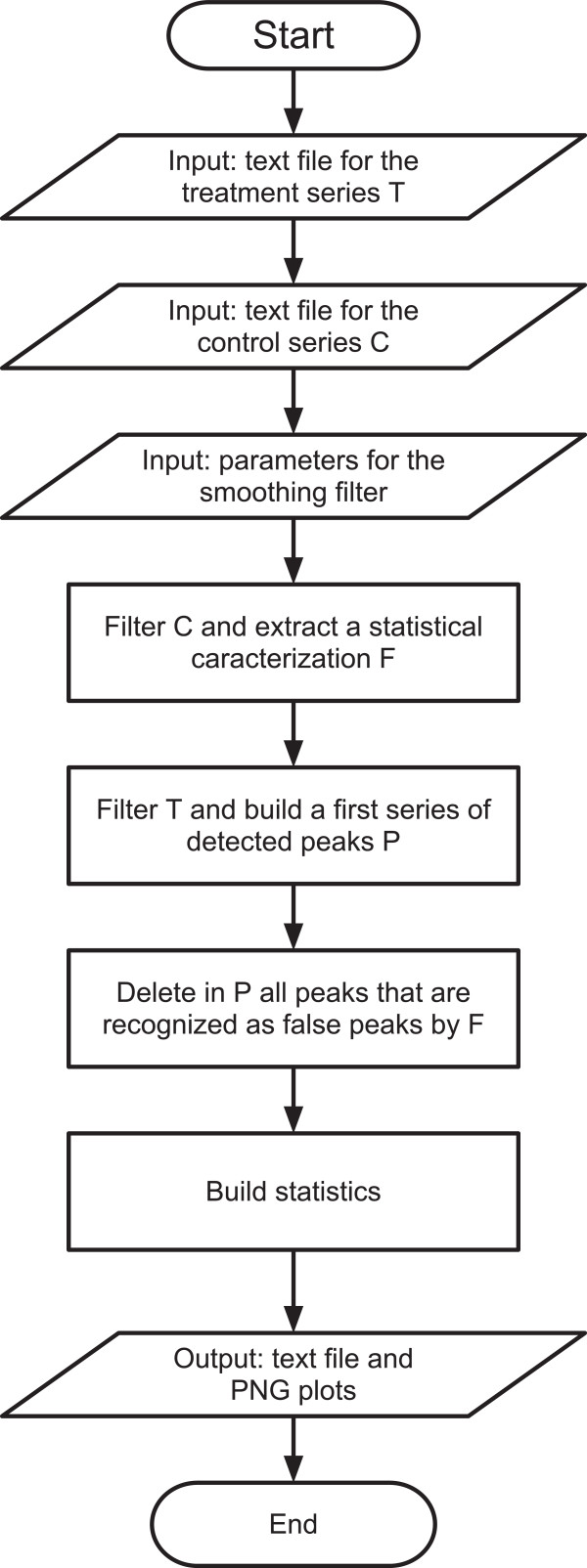
**CaSA software flow chart.** The chart resumes the main computational tasks performed by CaSA software. The required input data sets are the control and treatment time series files (see Supplementary Materials). All data are filtered for noise and a first analysis of the treatment series is made to detect putative peaks. Similarly, false positive peaks are identified in the control and used to characterize low-level oscillations that will be considered as noise. Putative peaks with such characteristics are then removed from the treatment time series. The software then computes the number of remaining peaks in each sample and measures the waiting times between adjacent peaks. All output data are presented in a text file that also contains basic statistical analyses, including the average peak number, the average waiting time and the number of samples where spiking can be detected. Lastly, PNG image files are generated presenting the plots of the original ratiometric data, the filtered trace, and indications of the detected peaks.

The computation flow, presented in Figure 1, proceeds as follows:

1. The treatment and control data sets, loaded by the user in the form of text files, are filtered for noise using a polynomial SG filter. The same filtering also provides point by point first and second derivatives. The filtering is controlled by the parameters listed below. In order to fine tune the analysis to the specific characteristics of different experimental systems, all of the parameters can be adjusted by the user via dialogue windows (Additional file 2 and Additional file 3).

(*w*) Width of the sliding measurement window. This must be an odd value, corresponding to the total number of time points that will be included in the filter window. If *n* points are considered before and after the filtered point (see Methods), then *w* = 2 *n* + 1. In general, a window width covering the maximum peak duration should be selected.

(*k*) Degree of the approximating polynomial curve. A default value of 5 (odd) was chosen to capture peak asymmetry, which is a constant feature in our spiking records, but different values can be entered by the user to best fit any peak shape.

(τ) Threshold for peak detection in the function *peak*_*c*_*(i).* This value determines the threshold in peak height above which an oscillation is identified as a peak rather than background noise. A default threshold is proposed by the software, but again the user can enter a value that provides the best signal/noise discrimination for a specific data set.

2. The application of the derived peak function to the filtered data is used to obtain an instantaneous indicator of peak occurrence. In the case of the control data set, where only background noise is recorded, this function identifies a number of ‘false’ peaks that will be used in step 3 to validate the significance of peaks detected for the treatment data.

3. The amplitude of each peak in the treatment data set is compared to the amplitude of ‘false’ peaks identified in the control data set. In brief, a peak is validated whenever its amplitude is higher than that measured for a defined percentage of the false peaks from the control data set. Such a percentage is defined as a quantile (ranging between 0 and 1) and can be chosen by the user in the dialog window where the *q* parameter is requested. A default value of 0.9 (corresponding to 90%) is automatically proposed by the software.

4. The following quantitative information is then obtained for each data set: number of peaks detected, peak-to-peak intervals (waiting time). Actively-responding cells are defined as those with a minimum of three peaks during the 30 min period of observation. Basic statistical analyses are carried out on the output data, including the average waiting time and the waiting time autocorrelation for each sample, as well as the average peak number in the actively responding cells from the whole data set.

**Figure 2:**
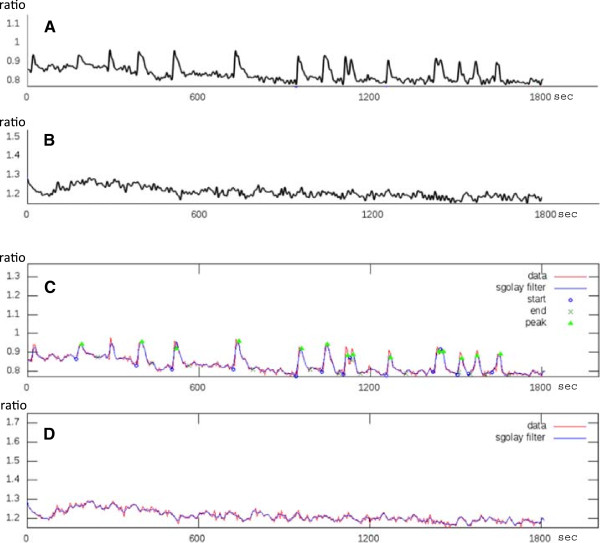
**Examples of data treatment by the CaSA software.** The graphs derived from the confocal microscope records (see Additional file 7) are shown in **A**, **B** (values on the Y axis represent the ratio of YFP to CFP fluorescence); the corresponding output plots are presented in **C**, **D**, where the original data are shown in red, while the blue lines correspond to the SG filtered curves. The maximum of each peak detected by the software is marked by a green triangle; the estimated starting and ending points are marked by a blue circle and a green cross respectively. **A**, **C** show a representative record from a cell treated with the AM fungal exudate; a typical spiking profile is visible and the CaSA software efficiently identifies each peak. **B**, **D** present the corresponding data from a control cell treated with sterile distilled water where no Ca^2+^ peaks are recorded.

After the analysis, the CaSA software outputs a series of plots as PNG image files, where the original ratiometric data, the SG filtered curve, and each of the detected peaks (including the starting points, maximum and ending points) are indicated. Figure 2A and B show representative profiles recorded from cells that were treated with either the fungal exudate or sterile distilled water (as control). Figure 2C and D show the results of CaSA software analysis of this data. The original time series (in red) is overlayed onto the SG filtered curve (blue) and the starting points, maximum and ending points are indicated for each detected peak.

An output text file is also created, containing all the results of the computational analysis (an example is presented as Additional file 4), such as the peak count for each sample, the identification of “active” vs. “inactive” cells (based on the presence of at least 3 peaks) and the autocorrelation analysis of peak-to-peak intervals (waiting times). The file includes a legend briefly explaining the meaning of each quantitative parameter.

The CaSA software is available for download with the supplementary data (Additional file 5).

### Case study 1: Ca^2+^ spiking responses to AM fungal exudates as a function of the Pi concentration in the fungal and plant growth media

With the aim of studying whether Pi availability can influence the early pre-infection steps of the AM interaction, and in particular the activation of Ca^2+^ signaling within the CSSP, we recorded nuclear Ca^2+^ spiking profiles in epidermal tissues of *M. truncatula* roots in response to germinated AM fungal exudates when both roots and fungus were grown under different phosphate conditions, as described in the Methods section and reported in Table 1.

**Table 1:** Root and fungal growth conditions used in the experiments for case 1

		**P l a n t**	
		**KH**_**2**_**PO**_**4 **_**35 μM**	**KH**_**2**_**PO**_**4 **_**3,5 mM**
**Fungus**	**--**	E_LP_-M_LP_	E_LP_-M_HP_
	**KH**_**2**_**PO**_**4 **_**3,5 mM**	E_HP_-M_LP_	E_HP_-M_HP_

Starting from the standard conditions [10] where *G. margarita* spores are germinated in distilled water (exudate low phosphate, E_LP_) and *M. truncatula* ROCs are grown on M medium (medium low phosphate, M_LP_), we considered three alternative cases where the Pi concentration is raised to 3,5 mM for either the plant (E_LP_-M_HP_) or the fungus (E_HP_-M_LP_) medium, or both (E_HP_-M_HP_).

**Figure 3:**
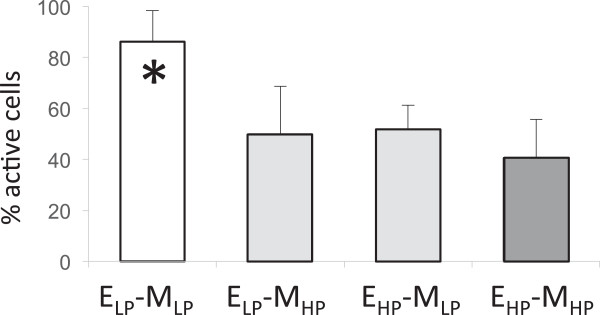
**Histogram describing the percentage of actively responding cells for each condition.** The percentage of responding cells in the E_LP_-M_LP_ (exudate low phosphate - medium low phosphate) condition is significantly different from all other treatments. N ≥ 4 roots per experiment; shown is average (± standard deviation [SD]); * indicates P value < 0.05 using unpaired Student t test when the data fit with a normal distribution or Wilcoxon- Mann–Whitney non parametric test in other cases.

**Figure 4:**
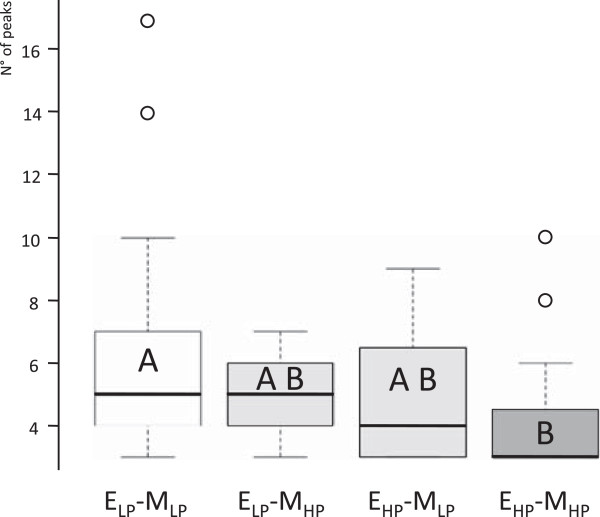
**Box-and-whisker plots of the peak number in each growth condition.** Statistical analysis indicates that when the fungus and the plant are both grown in high Pi (E_HP_-M_HP_ - exudate high phosphate/medium high phosphate) or low Pi (E_LP_-M_LP_ - exudate low phosphate/medium low phosphate), the observed Ca^2+^spiking patterns are significantly different from the two intermediate conditions (E_LP_-M_HP_; E_HP_-M_LP_). N ≥ 19 responding cells per experiment; The figure shows median peak numbers (black lines), 25% to 75% quartiles (boxes) and ranges (whiskers); letters indicate P value < 0.05 using nonparametric analysis of variance Kruskal-Wallis test with Dunn’s multiple comparison post test. Open circles represent extreme values.

By applying the CaSA software to our data we were able to calculate different parameters of the spiking response. Firstly, the percentage of ‘actively responding cells (defined as those displaying at least three peaks over the 30 min measurement period) was calculated for each treatment. As reported in Figure 3, statistical tests showed that the percentage of responding cells when both the plant roots and the fungus were grown under low phosphate conditions (the standard growth conditions used for in vitro AM development) was significantly higher compared to all other experimental combinations (p < 0.05, using unpaired parametric Student t test and Wilcoxon- Mann–Whitney non parametric test). The average number of peaks in the active cells, directly derived from the CaSA output file, was also used for comparison. Figure 4 shows that total peak averages were statistically higher when both roots and fungus were grown under low Pi (exudate low phosphate - medium low phosphate; E_LP_-M_LP_) and statistically lower when both roots and fungus were grown under high Pi (exudate high phosphate - medium high phosphate; E_HP_-M_HP_), confirmed by both Kruskal-Wallis (p = 5.4 • 10^-3^) and post-hoc tests (p = 3.9 • 10–3). Since the high Pi conditions used in our experiments did not affect fungal viability or spore germination rate (not shown), our results indicate that an inhibitory effect of Pi is already present during the earliest stages of the interaction that involve the activation of host nuclear Ca^2+^ spiking in response to fungal symbiotic signals present in germinating spore exudates. This inhibition is highlighted by the lower intensity of the Ca^2+^ spiking response (in terms of both the percentage of responding cells and average number of peaks) and is most striking when both the plant and the fungus are grown in high Pi media.

### Case study 2: Comparison of Ca^2+^ spiking profiles in response to AM fungal and rhizobial signals

**Figure 5:**
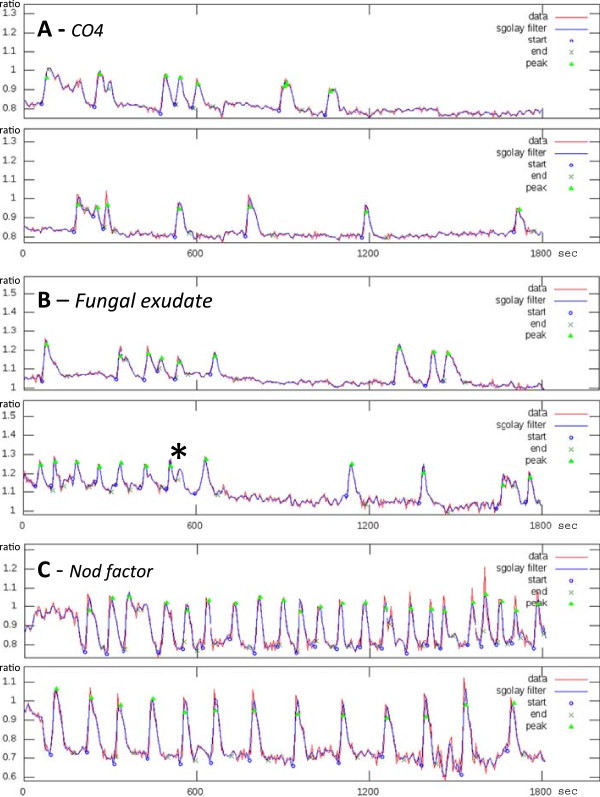
**Ca**^**2+ **^**spiking patterns induced by fungal and rhizobial signals.** Two representative examples of CaSA output files are presented following treatment with CO4 **(A)**, *G. margarita* spore exudate **(B)** or Nod factor **(C)**. Values on the Y axis represent the ratio of YFP to CFP fluorescence. The asterisk in B indicates an irregularly-shaped peak where the second rounded maximum is excluded from the CaSA computation. This is a typical case where manual peak identification would be uncertain, while automated analysis represents a reliable and repeatable method.

**Figure 6:**
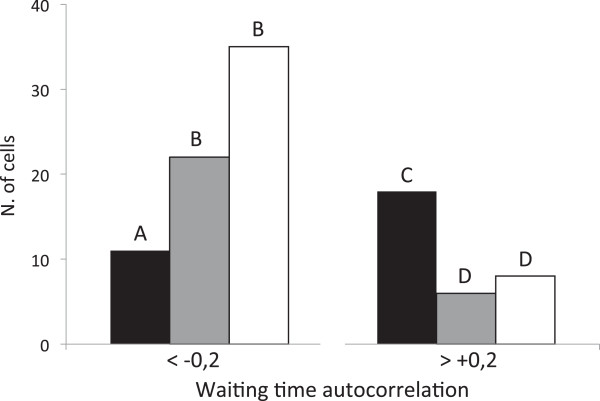
**Histogram illustrating the distribution of waiting time autocorrelation values in the spiking profiles induced by Nod factor (black), CO4 (grey) and fungal exudate (white).** The percentage of nuclei where autocorrelation value is significantly negative (< −0.2) or positive (> +0.2) are shown in the Figure. Waiting time autocorrelation values for CO4– and exudate-induced spiking display a maximum in the negative range, whereas Nod factor-induced spiking is characterized by a prevalence of positive autocorrelation values. Letters indicate statistically significant differences as highlighted by the Pearson χ^2^ test.

This case study aimed at identifying quantifiable features in the spiking patterns triggered by either Nod factors or CO4, the signal molecules that most effectively activate SNF- and AM-related nuclear Ca^2+^ signaling [13]. Since early SNF and AM responses focus on different cell types - root hairs versus atrichoblasts respectively - in this study we have compared root hair responses to Nod factors with atrichoblast responses to CO4. Several examples of typical spiking patterns observed in *M. truncatula* root epidermal cells in response to *S. meliloti* Nod factor or CO4 are shown in Figure 5. We found that CaSA was able to quantify significant and consistent differences between the two populations of spiking data sets. It turned out that the most striking differences concerned the time intervals between adjacent peaks (waiting time). By applying autocorrelation analysis to these values, the software identified a different periodicity, thus providing a quantitative parameter that discriminates the two spiking responses. As presented in Figure 6, the distribution of autocorrelation values is significantly different for CO4 and Nod factor treatments (Pearson χ^2^ test, p = 2.15 • 10^-6^). This indicates that the sequence of peak-to-peak intervals in response to Nod factor is relatively regular: long waiting times are in most cases followed by long waiting times and short waiting times are followed by short waiting times. By contrast, the CO4 treatment elicits spiking which is generally characterised by negative autocorrelation values, indicating that most cells alternate short and long waiting times.

For comparative purposes, we have applied the same CaSA analysis to a data set of Ca^2+^ spiking responses induced in the *M. truncatula* epidermis by *G. margarita* exudates prepared in the absence of phosphate (see E_LP_ condition in case study 1). Interestingly, the distribution of waiting time autocorrelation values is very similar to that for CO4-induced spiking, with no statistically significant difference (Pearson χ^2^ test, p = 0,01). This confirms the fact that short-length chitin oligomers can mimic the effect of AM fungal exudates in activating the CSSP and strengthens the proposal that these molecules are key signals in glomeromycete recognition by their hosts [13].

In conclusion, the CaSA software has allowed us to quantify significant differences in peak periodicity between the spiking patterns associated with AM fungal signaling (CO4 or fungal exudate) and rhizobial signaling (Nod factor).

## Discussion

The characterization of the molecular dialogues between symbiotic microbes and their plant hosts is of crucial importance in understanding the key recognition processes which precede successful associations. In the case of the endosymbiotic interactions between legumes and either glomeromycetes or rhizobia, research in many laboratories has to a large extent identified the genetic bases of the host mechanisms involved in the perception of fungal and bacterial signals, leading to the characterization of a common transduction pathway, the CSSP [30,31]. It has been proposed that distinct signatures exist for the nuclear Ca^2+^ spiking which is at the core of the CSSP [30,32]. Given the crucial role of Pi in the symbiotic nutrient trading between Glomeromycota and their host plants [33], growing interest is accumulating around the effects of available Pi on the establishment of AM [27,34,35]. The investigation of such processes has revealed a more complex picture than previously envisaged, suggesting the existence of a crosstalk in the plant perception of microbial signals and available nutrients.

Ca^2+^ spiking is the earliest known hallmark of symbiotic plant-microbe interactions and, as an increasing number of research groups focus on these topics, the variability and complexity of the observed spiking profiles is now becoming apparent. As a result, there is a crucial need for reliable tools that allow fast and reproducible analyses of spiking patterns. We propose the CaSA software as one such tool, and the examples we have chosen to illustrate its usefulness, targeted on two specific biological problems, and based on relatively large data sets, show that this approach can indeed identify and quantify distinct features of spiking profiles which can be used to define Ca^2+^ signatures.

### CaSA software performance

Compared to manual peak count and data analysis, the use of CaSA analysis resulted to be advantageous in terms of both time and reliability. Although a few file conversion steps are also required when using CaSA, the software dramatically reduced the time required for data analysis and figure preparation. We estimated that, excluding image acquisition and confocal data import to a datasheet file, 30 minutes were required for manual peak counting in a set of 50 nuclei. CaSA reduced this time to less than 5 minutes, saving more than 80% of operator time. Moreover, CaSA also provides a few statistical analyses that would further increase the required time if calculated manually.

Even if CaSA can be considered as a prototypal software in its present release, a major effort has been made to simplify usage while at the same time providing direct access to all of the major computational functions implemented in the automated spiking analysis process. For this, the user is presented with a limited number of dialog windows where the key parameters of the analysis can be entered. In all cases default values are proposed which correspond to those that were the most suitable for our studies. Nevertheless, by adjusting each parameter, the analysis can be fine-tuned to very diverse types of data. For example, by changing the degree of the approximating polynomial from 5 to 2, the software can better detect peaks where asymmetry is less pronounced or totally absent.

In addition to the generation of a text file containing all the results of the analysis, the last step in the CaSA process includes the rendering of a series of image files where the data are plotted. This graphical representation of the analysis has many applications. First of all it is of great help when adjusting the software parameters to better fit the spiking records. The identification of errors in the procedure is extremely easy since each peak detected can be directly checked with the overlaid original data. Secondly, a rapid screening of the output image files allows the identification of individual records which may contain instrument-related mistakes or other anomalies. Such records can be easily excluded from the subsequent computation runs by entering their corresponding progressive numbers into the final dialog window entitled ‘Samples to be excluded’. This option is also of use when a subset of plots is needed, for example in view of data publication.

As for all software, CaSA analysis is not error free. In our tests, false positive and negative peaks did occasionally appear even when the analysis parameters were adjusted to give optimal performance. Nevertheless, the impact of such rare errors did not modify the final interpretation of any of the global trends that we considered in our case studies. In addition, repeated trials where we compared manual and automated peak counting revealed that manual peak detection varies significantly from one operator to another, and even when the same operator repeats the counting under different conditions. By contrast, CaSA provides the obvious advantage of full repeatability of the analysis. A typical case where manual peak identification could be ambiguous can be found in the second plot of Figure 5B, where an irregularly shaped peak (marked by an asterisk) displays an initial maximum followed by a second weaker oscillation. In our tests, different operators identified either one or two peaks in this shape. By contrast only the first maximum is consistently identified as a peak by CaSA.

In conclusion we propose the CaSA software as a versatile tool that can be of help to obtain rapid and reliable analyses of diverse types of oscillatory data, including but not limited to Ca^2+^ spiking.

### Pi partially inhibits the presymbiotic dialogue in AM associations

A number of studies have analysed the physiological and molecular mechanisms that regulate the establishment of the AM symbiosis, highlighting the inhibitory effect of high phosphate availability on the extent of root colonization by AM fungi [26-28,34,36]. Here we have investigated whether the presence of high Pi in the growth media also affects earlier steps of the interaction, and in particular activation of the CSSP during the pre-symbiotic dialogue. Our analyses using CaSA suggest two distinct effects of high Pi concentration. Firstly, when both symbionts are independently grown under high Pi conditions, the root responsiveness to the fungal exudate is significantly reduced both in terms of the percentage of epidermal cells where the nuclear Ca^2+^ spiking signal is triggered and the total number of peaks recorded in responding cells. We interpret this as a partial inhibitory effect of Pi on the activation of the CSSP. This is a novel observation that suggests that at least part of the known inhibition of root colonization by AM fungi under comparable Pi concentrations [27,34] may be due to a reduced pre-symbiotic host response. In further support of this, high Pi has also been shown to moderately lower the expression levels of *Petunia* genes acting upstream of Ca^2+^ spiking in the CSSP [34].

Secondly, when high Pi is present only during AM spore germination there is also a significantly weaker plant response in terms of average peak number compared to the reference low Pi conditions. This suggests that high Pi levels not only affect the plant host, but are also perceived by the fungus. The ability of the AM fungus to detect extracellular Pi availability is perhaps not surprising, if we consider that glomeromycetes are particularly effective in Pi scavenging from soils. The implication that the AM fungus could in some way limit its own signaling to the host plant under Pi-rich conditions is extremely intriguing and certainly deserves further investigation. In line with this, [35] recently demonstrated that Pi availability modulates the expression of several fungal genes, including the Pi transporter GintPT in the AM fungus *Rhizophagus irregularis*. It should be underlined that our reference E_LP_-M_LP_ condition (35 μM Pi in the root culture medium and 0 μM in the spore germination medium) corresponds to the standard growth conditions that were found to be optimal for in vitro AM establishment [37,38]. The identical condition, where spore germination was induced in sterile distilled water, was also recently used to characterize the potential signaling molecules (chito- and lipochito-oligosaccharides) produced by the presymbiotic mycelium of AM fungi [10,13,20].

A recent publication [39] also investigated the effects of Pi availability on CSSP activation with a significantly different setup. Part of the study was focused on a later stage of the interaction - after hyphopodium contact. Secondly, fungal exudates used to induce Ca^2+^ spiking were only prepared in the absence of Pi and from a different fungal species (*Rhizophagus irregularis*): we currently cannot exclude that the complex connection between AM development and Pi availability is also dependent on the plant/fungus coupling.

We therefore conclude that Pi has a limited but significant effect on host plant perception/transduction of AM signals present in fungal exudates from *G. margarita*. This attenuated Ca^2+^ spiking response in the presence of high Pi appears to be due both to fungal and host partners. Since the optimal synthesis and secretion of strigolactones, the root-secreted molecules which stimulate pre-symbiotic hyphal branching in AM fungi [40], also requires Pi deficiency [29], we can now consider that high Pi functions as a general negative regulator of the molecular pre-symbiotic dialogue.

### Specific Ca^2+^ signatures for AM and SNF?

Ever since the discovery that both AM fungal and rhizobial signals are transduced by the CSSP in legumes [41], the question immediately arose as to the mechanisms by which two different signals could be transduced by the same pathway and activate different downstream responses [30]. Upstream of the CSSP, different receptors are probably involved in binding the respective AM fungal or rhizobial signals. Although a specific receptor for fungal signals has not yet been identified, this hypothesis is supported by the fact that putative Nod factor receptors (such as *MtNFP*) are not required for AM establishment [42]. Similarly, downstream of the CSSP, different transcription factors are involved in gene regulation during AM (*MtRAM1*[43]) and SNF (*MtNSP1*[44]). It has also been proposed that a central and essential component of the CSSP, the nuclear kinase MtDMI3, is differentially activated when binding Ca^2+^ ions and/or calmodulin [32]. This would indeed be in line with the generation of different calcium signals encoding AM- or SNF-specific information. The spiking profiles induced in SNF and AM have both been described as combining chaotic and stochastic elements [9,12], while part of the response-specificity could be related by the type of epidermal cell responding to either AM fungi or rhizobia: atrichoblasts and root hairs respectively [10].

The study reported here is based on the hypothesis of [9] concerning the presence of a stochastic component in AM- and SNF-related Ca^2+^ spiking. Stochasticity can be interpreted as evidence for the transmission of a message through a communication channel [45]. In this context, we have previously demonstrated the exponential distribution of waiting times in relation to the elapsed time from the initial stimulus [16], in agreement with the theoretically efficient use of a communication channel [45].

We therefore investigated the presence of a repetitive pattern in the sequence of peak-to-peak intervals, as a possible component of the calcium signature. Several techniques of pattern recognition can be found in literature which in most cases analyze the conformity of the experimental data to a predetermined pattern model [46]. Since we had no indication for a precise pattern model that could fit our case, we introduced the calculation of waiting time autocorrelation. This allowed the characterization two distinct trends in the spiking patterns associated with either AM fungi or rhizobia.

Based on the results presented in this article, we now propose that the explicit mechanism encoding the Ca^2+^-mediated message can be found in the variability of the waiting time. By introducing the calculation of waiting time autocorrelation we have been able to characterize two distinct trends in the spiking patterns associated with either AM fungi or rhizobia. In line with the hypothesis of [32], these results provide evidence for a quantifiable trait in nuclear Ca^2+^ signaling that can play a role in the capacity of the legume root epidermis to discriminate between the two symbionts.

## Conclusions

The CaSA software has been exploited in two different experimental contexts to further our understanding of the molecular/cellular signaling mechanisms underlying the establishment of symbiotic plant-microbe interactions. We therefore propose CaSA as an attractive platform to face so far unsolved questions concerning Ca^2+^ spiking signals and their modulation. Our findings suggest that AM fungal signal production could be limited in the presence of high inorganic phosphate levels, Since CO4 production is significantly enhanced by strigolactones [13], it would now be interesting to study strigolactone stimulation of the germinating AM spores as a function of different Pi concentrations. CaSA software has also revealed substantial, quantifiable differences between the Ca^2+^ spiking profiles triggered by either Nod factor or CO4, thus opening the question as to whether such differences remain when roots are treated with Myc-LCOs, whose chemical structures are more closely related to Nod factor [20].

## Methods

### Plant and fungal materials

*Medicago truncatula* genotype Jemalong A17 was used in this study. *Agrobacterium rhizogenes*-transformed root organ cultures (ROC) expressing the 35S:NupYC2.1 construct [23] were obtained according to [47]. NupYC2.1 is a cameleon probe that exploits Förster resonance energy transfer (FRET) to highlight variations in the level of Ca^2+^ concentration in the nucleoplasm. Transformed roots with a good level of nuclear fluorescence were selected three weeks after inoculation, decontaminated and grown as ROCs on minimal (M) medium at 25°C in the dark [10]. Since Ca^2+^ spiking responses to AM fungal signals are equivalent in composite plants and ROCs [10,13], we have only used the latter in this work. As summarized in Table 1, ROCs were grown in the presence of 35 μm KH_2_PO_4_ (M_LP_), a standard condition for in vitro AM development [48], or 3.5 mM (M_HP_), a concentration that does not affect root growth but inhibits root colonization by AM fungi [27]. *M. truncatula* ROCs were grown in vertically oriented Petri dishes to favour the development of a regular fishbone-shaped root system [48]. The AM fungus used in this study was *Gigaspora margarita* isolate BEG 34 (International Bank for the Glomeromycota, University of Kent, UK).

The spores were harvested from cultures of *Trifolium repens*, as described in [10]. In brief, spores were collected by repeated sieving under running water and stored at 4°C for one week to increase the germination rate.

### Root treatments

To record nuclear Ca^2+^ levels we applied the protocol described in [10]. Segments of primary roots of *M. truncatula* carrying one or two young laterals (2–4 cm in length) were placed in a 2-mm-thick microchamber on a microscope slide containing sterile distilled water. The water in the microchamber was then rapidly replaced by 100 μl of the treatment solution before starting image acquisition.

In our first case study, M_HP_ and M_LP_ roots were treated with fungal exudates. To produce the exudate (E), batches of 100 surface-sterilized *G. margarita* spores were placed in either 1 ml of sterile distilled water (E_LP_) or 3,5 mM KH_2_PO_4_ solution (E_HP_; see Table 1) and incubated for 7 d at 30°C in the dark to induce germination (germination rate > 90%). The germination medium was recovered by pipetting, concentrated 10-fold using a Lio5P lyophilizer (Cinquepascal, Milan, Italy) and stored at −20°C.

For our second case study, roots were treated with water solutions containing either 10-8 M CO4, 10^-8^ M Nod factor from *S. meliloti* or E_LP_. Nod factor treatments were performed on transformed roots from composite plants [13,47], since Nod factor responsiveness is absent in excised ROCs [49]. All records of nuclear Ca^2+^ spiking in *M. truncatula* root hairs in response to Nod factor were kindly provided by Mireille Chabaud and David Barker (LIPM, Toulouse, France). In both case studies, control treatments were included where the treatment solutions were replaced with sterile distilled water.

### Confocal microscopy and measurement of changes in nuclear Ca^2+^ levels

Confocal microscopy was used for all the FRET experiments. FRET-based detection and plotting of relative changes in nuclear Ca^2+^ levels were performed according to [23], by measuring the ratio of yellow fluorescent protein (YFP) to cyan fluorescent protein (CFP) signal intensity over time [50,51]. A 40× water-immersion objective was used and the pinhole was set to 6 Airy Units so that the thickness of the optical sectioning would embrace the average diameter of epidermal cell nuclei. 512 by 512 pixel frames were collected every 5 s for 30 minutes. Since glomeromycota and rhizobia target different cell types, analyses involving fungal signals were done on ROC atrichoblasts, while Nod factors responses were recorded in root hairs from composite plants. The exact number of biological replicates for each condition is reported in Additional file 6.

### Data preparation

The Leica Confocal Software was used to output the original values of YFP and CFP fluorescence intensity into text files (Additional file 7). Each file contained the values derived from five cells. Text files were then imported into a Microsoft Excel 2008 model file, where the ratio of YFP/CFP values was calculated for each time series (Additional file 8). The columns containing the ratiometric series were then exported to a new text file, to be used as an input file for the CaSA software. The CaSA software requires that both input files containing data series and control series are formatted as shown in Additional file 9. In brief, all time series must have the same length (in our case 360 time points); the series corresponding to each cell must be presented in tab-separated columns, where the first row contains the sample ID codes (e.g. “CELL_1”). As required for the Octave language, the first character of the text file must be #.

### Computational analysis

The analysis of time series displaying a spiking pattern (in our case a repetition of peaks in nuclear Ca^2+^ concentration) must address the following issues:

•removing fluctuations due to measurement errors, to better discriminate signals from background noise;

•identification of the instant in the timeline when each peak event occurs, in order to allow automated operations such as counting the number of peaks and measuring peak-to-peak intervals;

•characterization of trends, to identify conserved traits in a population of records; automatically producing statistical analyses of the data obtained.

The first requirement implies the application of a smoothing filter to the original data. Such a smoothing process must level out background oscillations due to instrumental errors, so that only the major signal oscillations (peaks) stand out and can subsequently be identified and analyzed by the software. To this aim, the low-pass Savitzky-Golay filter family [52] is of common use and has demonstrated particularly efficient on noisy data sets [53]. In brief, rather than having their properties defined in the Fourier domain and then converted to the time domain, the Savitzky-Golay filters are derived directly from a particular formulation of the data smoothing problem in the time domain. This class of filters also provides an estimate of point to point derivatives along a curve, allowing the assessment of analytical properties.

The operating assumptions for the use of the method underlying these filters are: 1) the data to be processed are recorded at fixed and uniform intervals; 2) the phenomenon analyzed can be represented by a continuous curve.

The method takes into account a sliding window of measurements embracing the point *f*_0_ on which the filtering is applied, and two finite sequences of points of length *n* respectively preceding and following *f*_0_.

$$f{-}_{n},f{-}_{n+1}\cdot \cdot \cdot ,f{-}_{1},\cdot \cdot \cdot ,f_{n-1}f_{n}$$

A polynomial curve of degree *k* is calculated on this window of points to approximate the trend of the quantity with a continuous curve. Since in general 2 *n* + 1 > *k* +1 the method of least squares can be used. The filtered value of *f*_0_ is given by the value of the fitting curve in position 0 in relation to preceding values *f*_− *n*_, *f*_− *n* + 1_, · ··, *f*_− 1_ and following values f_1_, · ··, f_n − 1_f_n_.

From the polynomial approximation, an estimate of the first and second derivatives in position *i* can also determine the presence of a peak considering the conditions:

1. *f'i* = 0

2. *f''i* < 0

Other conditions, such as threshold exceeding and asymmetries of trends can then be integrated with the above conditions to form precise patterns of merit characterizing the peaks. For example, the expression

$$\mathrm{peak}\left(i\right):=|f_{i}>\delta |\times |-\xi <f{'}_{i}<\gamma |$$

with

|·| = 1 if true, 0 if false

expresses the presence of a peak once the δ ξ γ parameters (which characterize value, trend and symmetry respectively) are fixed. In the practice, the above function has been implemented with a continuous function *peak*_*c*_*(i)* using the product of absolute values instead of the logic function. A threshold τ has then been introduced to discriminate peaks from background oscillations: *peak (i)* = 1 if and only if *peak*_*c*_*(i)* > τ.

By analyzing the trend in the neighborhood of *f*_0_, it is also possible to estimate the peak event start (*t*_*s*_) and end (*t*_*e*_), by scrolling the series of the derivatives of each peak from left to right. From this temporal information and values detected, the *intensity* of a phenomenon can be evaluated with the integration:

$$I_{0}=\int_{t_{s}}^{t_{e}}f\left(t\right)\mathrm{dt}$$

which gives the area under the curve *f (t)* from *t*_*s*_ to *t*_*e*_ This value allows the comparison of peaks from different time series. In our case, considering a series from the control data set, the above formulation allows the determination of a value of intensity below which a peak is not to be considered significant and must therefore be excluded from computation in the treatment data set.

## Competing interests

The authors declare that they have no competing interests.

## Authors’ contributions

GR carried out all the experimental work, contributed to developing the automated analysis and the CaSA software; SS and ES developed the automated analysis and the CaSA software; PB participated in conceiving and coordinating the study; AG participated in designing and coordinating the study and drafting the manuscript. All authors contributed to the final version of the manuscript, which they have read and approved.

## Supplementary Material

Additional file 1 **Pipeline of CaSA usage.** The flow chart schematizes file preparation and data flow through the CaSA software. The steps leading to input data file production are representative of our experiments, but CaSA only requires the input data files, independently of the upstream experimental setup. Click here for file

Additional file 2 **CaSA usage video.** The video (in MPEG4 format) briefly explains CaSA installation procedure and basic use of the software. Click here for file

Additional file 3 **Effects of CaSA parameter variations on peak identification.** The figure shows how five different settings of the CaSA parameters (changes are highlighted in red) impact on peak identification (green triangles) in the resulting output plots. The optimal conditions for our experimental data set (which are proposed as a default by the CaSA software) are presented in the central box. For example, the width of the sliding measurement window (w) is set to 9 sampling points: with our sampling interval of 5 seconds, the window covers 45 (9 x 5) seconds, which is comparable with the average peak duration in our records. Longer or shorter windows can be chosen to best fit data sets with longer or shorter peak durations, respectively. Two examples are provided for each setting: one plot where the baseline shows a steep slope and peaks are more irregular (top), and one where peaks are very evident (bottom). While peaks are always identified correctly in the bottom plots, the fine tuning of CaSA parameters *w*, τ and *q* is crucial to optimize peak recognition in the top plots. For example, after increasing the *w* value to 13 (bottom box), changing τ from 1 to 2 or reducing *q* to 0.7, one or more peaks are left unmarked (asterisks). Click here for file

Additional file 4 **CaSA output file.** This is an example of the output text file produced by CaSA. The file displays the results of automated analysis for each sample. Sample IDs are reported in the first row, followed by statistical analyses on waiting times, peak numbers, percentage of responsive samples in the dataset (displaying more than 2 peaks during the recorded period). Click here for file

Additional file 5 **CaSA software.** This compressed (zip) file contains the CaSA software and associated files. All files should be downloaded to the same directory for the software to work. The software can be run from the terminal using the command line ./CaSA.m or by double-clicking on the CaSA.m icon from the file manager. In this case the file must be previously set as executable in the file properties. Input files should also be placed in the same directory as CaSA. Click here for file

Additional file 6 **Table summarizing the number of biological replicates used to generate the data for this research.** Since glomeromycota and rhizobia target different cell types, fungal signals effects were analysed in atrichoblasts from root organ cultures (ROC) and Nod factors (NF) effects in root hairs from composite plants. Click here for file

Additional file 7 **Original data file exported by the Leica Confocal Software.** This text file is the standard output obtained from the LCS software by exporting 30 min recordings of the fluorescence intensity (stack profile function) in five epidermal nuclei from *M. truncatula* roots treated with the fungal exudate. Click here for file

Additional file 8 **Spreadsheet calculating FRET intensity.** This Microsoft Excel file was used to calculate the FRET values (sheet 1) from the original data exported by the Leica Confocal Software (sheet 2, Additional file 7). Click here for file

Additional file 9 **CaSA input file.** This is the input file type required by the CaSA software. Two files are requested, one for the treated samples and one for the controls, where only background noise is recorded. Each column in the file contains the sample ID number and the FRET values as obtained by the Microsoft Excel spreadsheet (Additional file 8). Click here for file
