# Supplementary material for: Automated analysis of calcium spiking profiles with CaSA software: two case studies from root-microbe symbioses
Source: BMC Plant Biol. 2013 Dec 26;13:224. doi: 10.1186/1471-2229-13-224 (PMC3880239; doi:10.1186/1471-2229-13-224)
Supplement: Additional file 1 — Pipeline of CaSA usage. The flow chart schematizes file preparation and data flow through the CaSA software. The steps leading to input data file production are representative of our experiments, but CaSA only requires the input data files, independently of the upstream experimental setup. [file 1471-2229-13-224-S1.pdf]

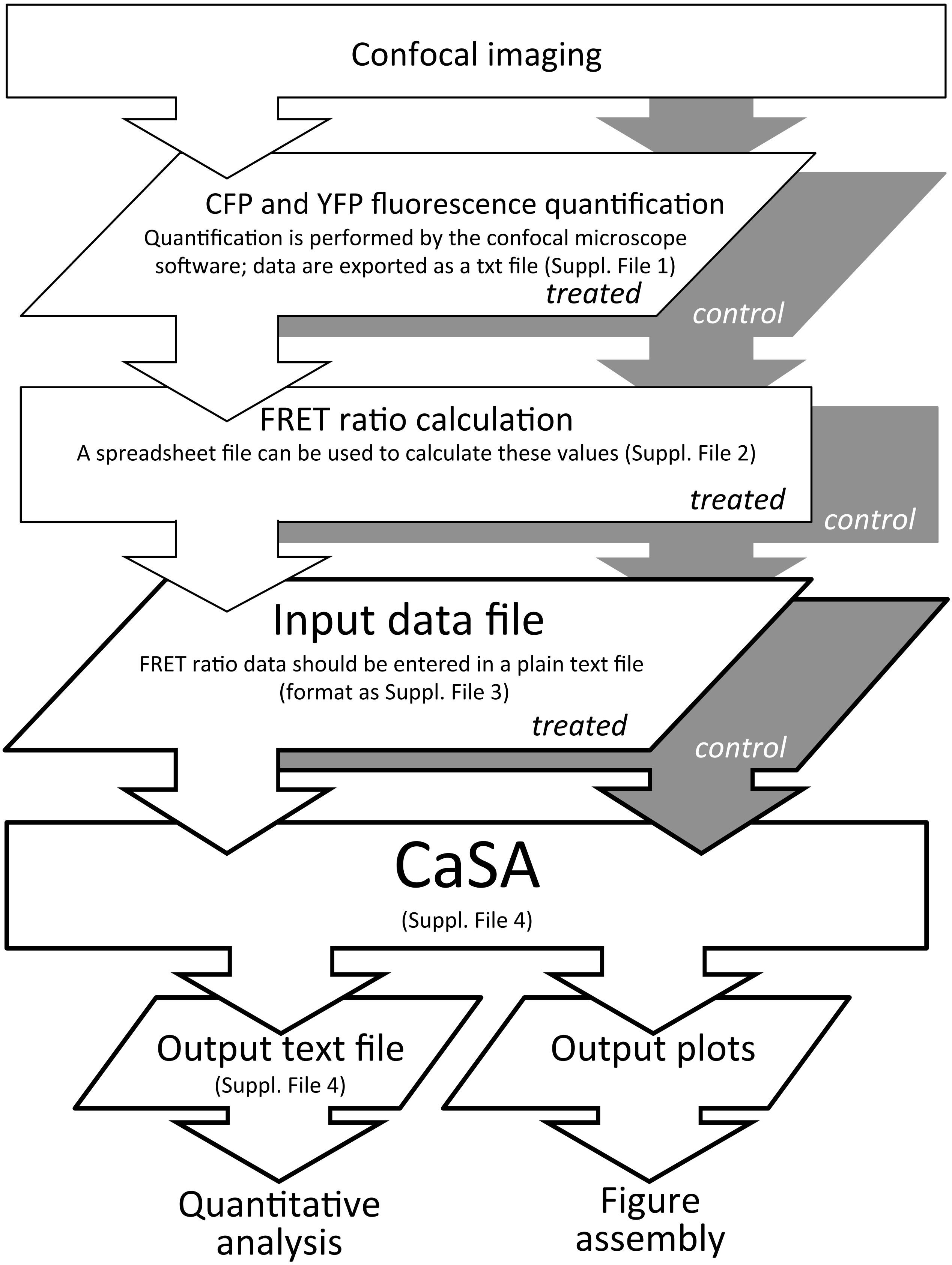

Supplementary File 8. Pipeline of CaSA usage. The flow chart schematizes file preparation and data flow through the CaSA software. The steps leading to input data file production are representative of our experiments, but CaSA only requires the input data files, independently of the upstream experimental setup.
