# Supplementary material for: Automated analysis of calcium spiking profiles with CaSA software: two case studies from root-microbe symbioses
Source: BMC Plant Biol. 2013 Dec 26;13:224. doi: 10.1186/1471-2229-13-224 (PMC3880239; doi:10.1186/1471-2229-13-224)
Supplement: Additional file 3 — Effects of CaSA parameter variations on peak identification. The figure shows how five different settings of the CaSA parameters (changes are highlighted in red) impact on peak identification (green triangles) in the resulting output plots. The optimal conditions for our experimental data set (which are proposed as a default by the CaSA software) are presented in the central box. For example, the width of the sliding measurement window (w) is set to 9 sampling points: with our sampling interval of 5 seconds, the window covers 45 (9 x 5) seconds, which is comparable with the average peak duration in our records. Longer or shorter windows can be chosen to best fit data sets with longer or shorter peak durations, respectively. Two examples are provided for each setting: one plot where the baseline shows a steep slope and peaks are more irregular (top), and one where peaks are very evident (bottom). While peaks are always identified correctly in the bottom plots, the fine tuning of CaSA parameters w, τ and q is crucial to optimize peak recognition in the top plots. For example, after increasing the w value to 13 (bottom box), changing τ from 1 to 2 or reducing q to 0.7, one or more peaks are left unmarked (asterisks). [file 1471-2229-13-224-S3.pdf]

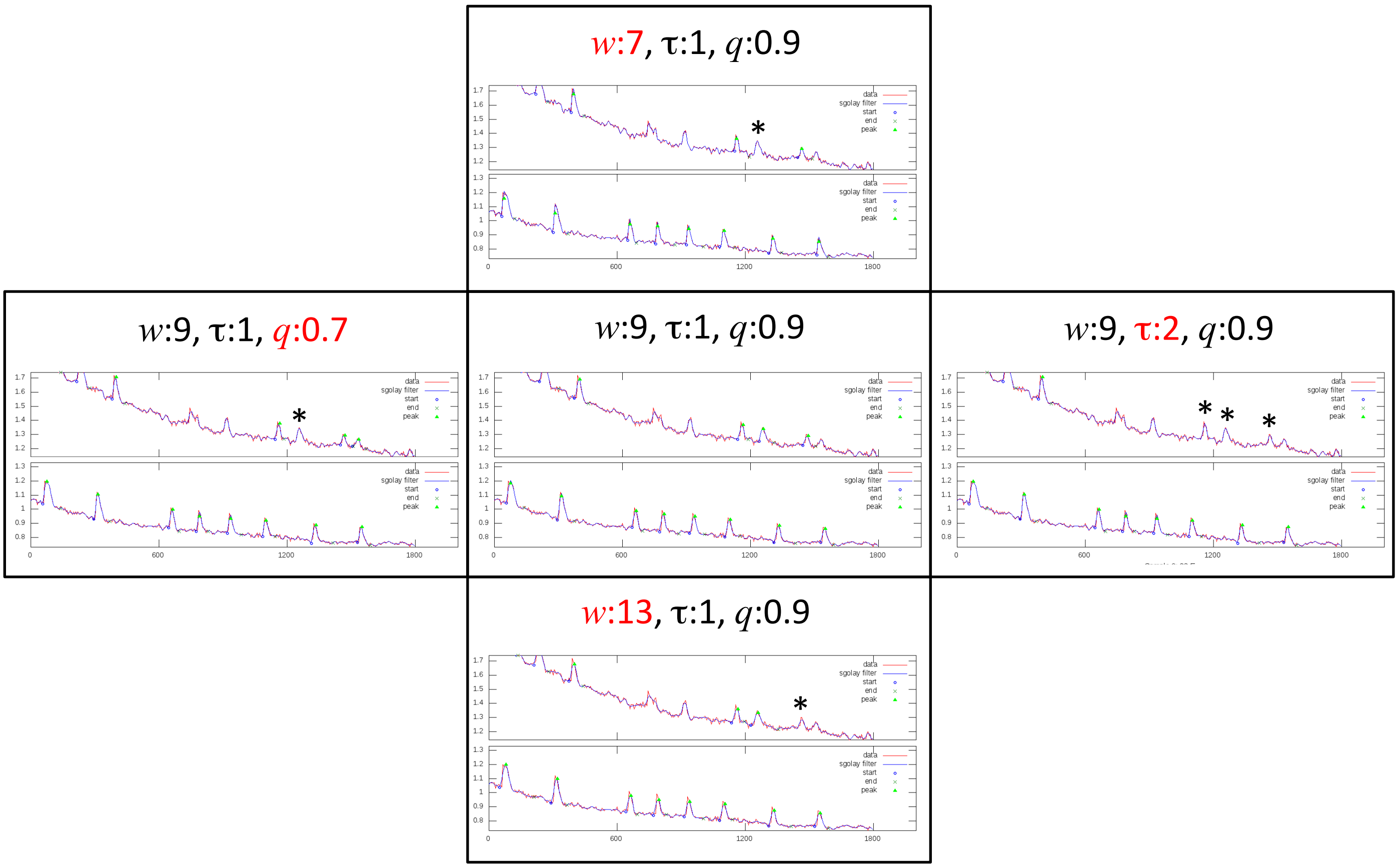

Supplementary Figure 7. Effects of CaSA parameter variations on peak identification. The figure shows how five different settings of the CaSA parameters (changes are highlighted in red) impact on peak identification (green triangles) in the resulting output plots. The optimal conditions for our experimental data set (which are proposed as a default by the CaSA software) are presented in the central box. For example, the width of the sliding measurement window (w) is set to 9 sampling points: with our sampling interval of 5 seconds, the window covers 45 (9 x 5) seconds, which is comparable with the average peak duration in our records. Longer or shorter windows can be chosen to best fit data sets with longer or shorter peak durations, respectively.

Two examples are provided for each setting: one plot where the baseline shows a steep slope and peaks are more irregular (top), and one where peaks are very evident (bottom). While peaks are always identified correctly in the bottom plots, the fine tuning of CaSA parameters *w*, τ and *q* is crucial to optimize peak recognition in the top plots. For example, after increasing the w value to 13 (bottom box), changing τ from 1 to 2 or reducing q to 0.7, one or more peaks are left unmarked (asterisks).
