# Supplementary figures and images for: Automated analysis of calcium spiking profiles with CaSA software: two case studies from root-microbe symbioses
Source: BMC Plant Biol. 2013 Dec 26;13:224. doi: 10.1186/1471-2229-13-224 (PMC3880239; doi:10.1186/1471-2229-13-224)

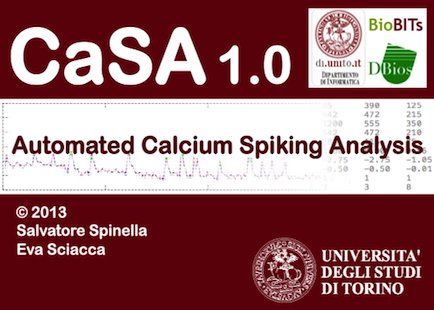

Supplement: Additional file 5 — CaSA software. This compressed (zip) file contains the CaSA software and associated files. All files should be downloaded to the same directory for the software to work. The software can be run from the terminal using the command line ./CaSA.m or by double-clicking on the CaSA.m icon from the file manager. In this case the file must be previously set as executable in the file properties. Input files should also be placed in the same directory as CaSA. [file 1471-2229-13-224-S5.zip › CaSA/splash2.png]
