# Supplementary material for: Automated analysis of calcium spiking profiles with CaSA software: two case studies from root-microbe symbioses
Source: BMC Plant Biol. 2013 Dec 26;13:224. doi: 10.1186/1471-2229-13-224 (PMC3880239; doi:10.1186/1471-2229-13-224)
Supplement: Additional file 6 — Table summarizing the number of biological replicates used to generate the data for this research. Since glomeromycota and rhizobia target different cell types, fungal signals effects were analysed in atrichoblasts from root organ cultures (ROC) and Nod factors (NF) effects in root hairs from composite plants. [file 1471-2229-13-224-S6.pdf]

Supplementary File 9. Table summarizing the number of biological replicates used to generate the data for this research. Since glomeromycota and rhizobia target different cell types, fungal signals effects were analysed in atrichoblasts from root organ cultures (ROC) and Nod factors (NF) effects in root hairs from composite plants.

	N° of roots	Material	N° of cells	Cell type
E_{LP}-M_{LP}	5	ROC	55	atrichoblasts
E_{LP}-M_{HP}	5	ROC	46	atrichoblasts
E_{HP}-M_{LP}	5	ROC	64	atrichoblasts
E_{HP}-M_{HP}	5	ROC	46	atrichoblasts
NF	6	composite plant	19	root hairs
CO5	4	ROC	32	atrichoblasts
